# Synthesis and Structural Characterization of Biofuel From Cocklebur sp., Using Zinc Oxide Nano-Particle: A Novel Energy Crop for Bioenergy Industry

**DOI:** 10.3389/fbioe.2020.00756

**Published:** 2020-09-04

**Authors:** Kifayat Ullah, Hammad Ahmad Jan, Mushtaq Ahmad, Anwar Ullah

**Affiliations:** ^1^Department of Biosciences, COMSATS University Islamabad, Islamabad, Pakistan; ^2^Biofuel & Biodiversity Lab, Department of Plant Sciences, Quaid-i-Azam University, Islamabad, Pakistan

**Keywords:** cocklebur energy crop, novel none-edible feedstock, biofuel synthesis, experimental protocol optimization, chemical and structural characterization

## Abstract

This study is reporting the biofuel synthesis and characterization from the novel non-edible feedstock cocklebur seeds oil. The Cocklebur crop seeds oil was studied as a potential source for biofuel production based on the chemical, structural and fuel properties analysis. The oil expression and FFAs content in cocklebur crop was reported 37.2% and 0.47 gram KOH/g, using soxhlet apparatus and acid base titration method, respectively. The maximum conversion and yield of the cocklebur crop seeds non-edible oil to biofuel was pursued 93.33%, using transesterification process. The optimum protocol for maximum conversion yield was adjusted: 1:7 oil-methanol molar ratios, ZnO nano-particle concentration 0.2 gm (w/w), reaction temperature 60°C, and reaction time 45 min, respectively. ZnO nano-particle was prepared by a modified sol-gel method, using gelatin and the particle was XRD, TEM, XPS, and UV-vis spectroscopies. Qualitatively, the cocklebur crop synthesized biofuel was quantified and structurally characterized by GC/MS, FT-IR, NMR, and AAS spectroscopies. Quantitatively, the fuel properties of cocklebur crop biofuel was analyzed and compared with the international ASTM and EN standards.

## Introduction

The fossil fuel resources particularly petroleum, coal and natural gas are the major energy sources all over the world. Due to increasing reliance of human over fossil energy sources resulted in the increase of assets’ exhaustion rate and global warming threats. Therefore, the alternate fuel resources are the necessity of time to be found in order to reduce the reliance over conventional fuels and to decrease as well the greenhouse gas emissions. Biomass derivative fuels could be one of the feasible results, since they are renewable and go about as carbon sink alternative (Alok et al., 2017; Zhangcai et al., 2018).

Among the various renewable sources of energy, biofuel (biodiesel) has picked-up great attention over petrodiesel. The possible source options are animal fats or plants oils through the process of transesterification reaction. The basic technique is the treatment of alcohol (methanol or ethanol) with fats or oils in the presence of acidic, alkaline, or enzymatic catalysts to synthesize mono-alkyl ester (Marcos et al., 2018; Wai et al., 2018). However, the use of plants’ oils as a source rivals its use as a well spring of nourishment. Continuing the above discussion, for biodiesel production low quality feedstocks must be utilized as crude resources. In this regard, animal fats, waste frying oils and non-edible oils appear to be the most suitable crude resources. However, most of these resources have having a high free fatty acid contents which require the specified two steps method (first esterification and then transesterification) because high free fatty acid contents are not suitable for transesterification, unless, their free fatty acid contents is reduced to <1%, which compares to 1 mg KOH/g of potassium hydroxide. While, the maximum amount of alkaline catalyst cause the form of soaps; henceforth, diminishing the biodiesel yield (Živković and Veljković, 2018).

Currently, research has been in progress on new feedstocks for sustainable fuels and similarly to enhance the conversion efficiency of methyl esters, using unique nano-particles. These days, nano-particles have been gained more entrust in the research field since last time due to its applications medicine industry, bio-imaging sector, sensors equipment’s etc. (Mitsudome et al., 2008; del Río et al., 2016; Kravets et al., 2016; Mesch et al., 2016; Silva et al., 2016; Tel-Vered et al., 2016). The maximum surface area of nano-particles with optimum energy define the catalytic application, whereas, some have been reported as the unique catalysts for a known chemical reactions like nucleation of CNTs, CO oxidation, dehydrogenation and many more (Ma and Hanna, 1999; Demirbas, 2006; Veljković et al., 2006; Ahmad et al., 2011). In the proposed work, ZnO particle was designed to check the status of nano-particle in cocklebur crop oil conversion. Results had shown an optimum catalytic efficiency in cocklebur crop methyl esters production.

**FIGURE 1:**
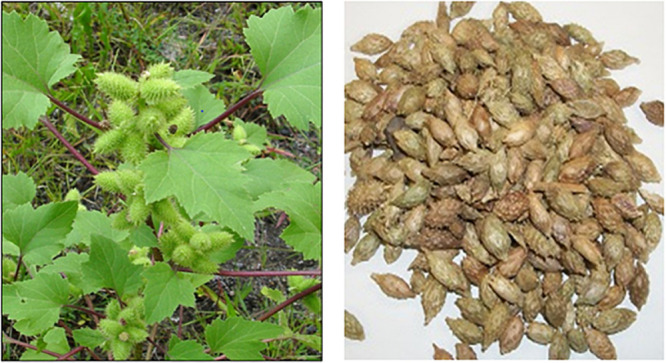
**(A,B)** Field Photograph of Energy Crop Cocklebur Sp. (*Xanthium strumarium* L.) and Dried Seeds.

Cocklebur crop (*Xanthium strumarium* L.) is an annual herb (Figure 1A), it grows up to the height of 20−120 cm. In plant, the nodal spines are absent. Petiole ranges from 3.5 to 10 cm in length. The leaves are median cauline, ovate to deltate form 9−25 cm. The leaves are densely scabrid on both sides. At base side, the leaves are shallowly cordate to broadly cuneate and the margin is irregularly dentate in structure. The apex is acute; Capitula are monoecious. The male capitula in terminal end is umbels; oblong to lanceolate. The calyx is about 2.2 mm in length. The outer paleae are oblong to lanceolate and the inner paleae absolatly lanceolate. Corolla is white in color, tubular, usually 2.5 mm in length. The female capitula are axillary; oblong-lanceolate. The inner bracts connate with outer paleae, and the burs are sessile, oblong, ellipsoid and ovoid up to 10−18 × 6−12 mm in length. The flowering period stared from July to August and started fruiting (Figure 1B) in September to October (Acharya et al., 2019).

It belongs to family Asteraceae and flourishes commonly in anxious, along lakes margins, streams sides, shores sides, marshes banks, banks of sloughs, railway tract sides and roads sides. It grows over different soil types from sands to heavy, however grows sound in compact sandy soil with less organic fertility and high water contents. The soil pH which sustains the plant is ranging from 5.2 to 8.0. The plant can tolerate frequent flooding and saline conditions. It is available almost everywhere and is being considered a wild plant. It is one of the cheap, economic and indigenous sources for the quantitative and qualitative production of biodiesel.

Our literature survey show that there is no comprehensive work reported yet on this novel non-edible oil yielding energy crop species, particularly on the production of biofuel (biodiesel) for bioenergy industry. The present study aims to produce a quantitative and qualitative biodiesel from the proposed novel feedstock seeds oil through the double step process called esterification followed by transesterification, using acid catalyst H_2_SO_4_ and ZnO nano-particle, respectively. Thereafter, to know the structure and nature of the novel non-edible feedstock synthesized biodiesel, possible analytical techniques: GC/MS, FT-IR, NMR and AAS and various fuel properties tests are designed to be carried out in laboratory for the authenticate findings data and documentation.

## Materials and Methods

In the proposed work, chemicals i.e., Sulfuric acid 98% and base catalyst Zinc oxide (ZnO) were used in esterification followed by transesterification reaction, accordingly. The Anhydrous methanol, Iso-propanol, Molecular sieve, Oxalic acid, Petroleum ether etc. were used consequently in the designed experimentation, accordingly. The entire chemicals were purchased from Merck and Scharlau companies were used without any alteration. The cocklebur crop oil was extracted from the collected dried seeds of plant, which were collected during field visit from various part of the country.

## Experimental Section

### Energy Crop Seeds Oil Extraction

The oil extraction from cocklebur crop dried seeds was the first step in biofuel production. The oil was extracted by two techniques i.e., Chemical extraction (Soxhlet apparatus) and Mechanical extraction. Prior to oil extraction, to eradicate dust the seeds were washed by distilled water and then were dried at room temperature under control laboratory environment.

#### Chemical Extraction (Soxhlet Apparatus Extraction)

Chemical extraction of oil was done for the determination of the oil expression in cocklebur crop seeds. The procedure was completed categorically in five sub-stages; started from grinding of seeds, extraction of oil through ether solvent, filtration, distillation and purification, respectively. Initially, 15 g of cocklebur crop seeds was taken and was desiccated at 60°C for 2−3 h in oven. The seeds were then smashed into fine powder by mortar and pestle. The fine powder of fixed quantity of 10 gm was taken in soxhlet apparatus in thimble. In the Soxhlet round bottom flask petroleum ether as a solvent of about 250 mL was taken. The temperature of soxhlet was adjusted at 60°C. At constant temperature, vapors started in the column of soxhlet apparatus toward the condenser. On condensation, the vapors condensed and fall down in the thimble. The oil dissolved in the solvent seeps into siphon tube through the pores of thimble, and gathered in the round bottom flask. This process was kept continued for 8 h under controlled environment. The extracted oil distilled at 60°C for 8 h to remove the solvent. To determine the extracted oil percentage the following equation was used (Ullah et al., 2015c).

(1) O⁢i⁢l⁢C⁢o⁢n⁢t⁢e⁢n⁢t⁢s⁢P⁢e⁢r⁢c⁢e⁢n⁢t⁢a⁢g⁢e;W=(W+3W)1W2

*Where*, *W*_1_ = *Weight of empty glass flask;*

*W*_2_ = *Weight of fine powder sample;*

*W*_3_ = *Glass flask and extracted oil weight;*

*W*_4_ = *Weight of extracting oil weight.*

#### Mechanical Oil Extraction

To obtain oil in large quantity oil was extracted by mechanical method through electric oil expeller (KEK P0015, 10127 Germany). This crude extracted oil was then filtered out to remove impurities and was stored in glass jar for further laboratory experimentation work.

### Determination of FFA Contents

The cocklebur crop seed oil was tested for free fatty acid contents through acid-base titration method. In the conical flask oil sample of 1 mL was taken and added to it Iso-porpanol of 10 mL, and phenolphthalein indicator 2*−*3 drops. This sample of oil was then titrated against KOH (potassium hydroxide), untill the color of solution was changed to pink (Mesch et al., 2016). The crude oil free fatty acid content was calculated through the equation 2.

(2) F⁢r⁢e⁢e⁢F⁢a⁢t⁢t⁢y⁢A⁢c⁢i⁢d⁢s%=(A-B)×C×100/V

Where; A = Volume of Potassium hydroxide used during sample titration;

B = Volume of Potassium hydroxide used during blank titration;

C = Concentration of Potassium hydroxide (g/L);

V = Volume of oil sample.

### ZnO Nano-Particle Synthesis

The ZnO nano-powder was produced via amended sol-gel procedure through gelatin. Initially, 5 g product at first, the solution of gelatin was produced by adding 10 gm gelatin in 150 mL deionized water at 60°C. Whereas, under protocol, zinc nitrate was dissolved in a specific deionized water at room temperature. Both the solutions mixed up and stirred for 8−10 h at 80°C constant temperature. In result, the resin was calcined with various temperature, ranging from 500 to 600 and 700°C, respectively to get the proposed ZnO nano-powder. The morphological and structural analysis ZnO was characterized by XRD, TEM, XPS, and UV-vis spectroscopies.

### Biofuel Synthesis

The dried seeds of cocklebur crop were collected from different sites of the country by using various field trips. The collected seeds cleaned through distilled water for removing the impurities and dried at 60°C in oven. The dried seeds were subjected for oil extraction using KEK P0015, 10127 (Germany) model of mechanical electric oil expeller. The extracted oil was filtered through filter paper no. 42 (whatmann) for removing the impurities and stored in a glass jar at room temperature. The free fatty acid contents of extracted oil were resolute through aqueous acid-base titration technique (Knothe, 2010; Pengmei et al., 2010). *The two step process a*cid catalysis followed by base catalysis was carried out for the synthesis of biodiesel. Initially, the oil pre-heated up to 120°C to evaporate the water and degraded to diglycerides, monoglycerides, respectively. The temperature of oil reduced up to 60°C and mixed the methanol with optimum ration of 1:11 Oil-Molar ration and 1 mL sulfuric acid as an acid catalyst and precedes the reaction in 2L 3 necked round bottom glasses flask, which was equipped with sampling outlet, reflux condenser, magnetic stirrer and thermometer. While, after esterification process, the esterified oil treated with optimum 1:7 Oil-Methanol molar ratio using base catalyst Zinc oxide (ZnO), temperature 60°C and reaction time; 45 min, respectively (Gelbard et al., 1995; Kibler and Al-Shakran, 2016; Shi et al., 2016). All the experiments/reactions were performed in triplicate, accordingly.

#### Variables Impact on Conversion Reaction

To study the effects of different variables effect i.e., methanol oil molar ratio, catalyst concentration, temperature variation and reaction time a series of experiments were conducted. The methanol to oil molar ratios were used as 1:5, 1:7, 1:9, 1:11, 1:13; catalyst concentration started from 0.50, 0.75, 1.0, 1.25, 1.50%, temperature variation; 15, 30, 45, 60, 70°C and reaction time 50, 55, 60, 65, and 70 min, respectively. The conversion reaction and biodiesel yield was profoundly affected by the following variables discussed above.

### Fuel Properties Analysis

The present work covers and determines the synthesized biofuel fuel properties analysis. The following fuel characteristics i.e., Density, Kinematic Viscosity, Sulfur Contents, Pour Point, Flash Point, Cloud Point, Calorific Value, Acid Number, Cetane Number, Phosphorus Contents, Calcium, Potassium, Magnesium, and Sodium were studied and matched with the standards of EN 14214 and ASTM D 6751.

### Chemical and Structural Characterization of Ccocklebur Crop Biofuel Using GC/MS, FT-IR, ^1^H, ^13^C NMR, and AAS Spectroscopic Analysis

The confirmation of the level of conversion of cocklebur crop seed oil to biofuel and to determine the configuration of glycerol, free fatty acids, metals concentrations, alkyl hydrocarbons and un-reacted alkyl alcohol etc. is a paramount. To figure out quantitatively and qualitatively, all these distinctive analytical techniques: GC/MS, FT-IR, ^1^H, ^13^C NMR, and AAS were used, respectively (Begum et al., 2016).

#### Quantification of Biodiesel by GC/MS Analysis

The quantification and characterization of cocklebur crop biofuel was determined by GC (GC–6890N Model) coupled with MS [MS–5973 Model, Mass Selective Detector (MSD)]. A capillary column DB-5MS (30 m × 0.32 mm, 0.25 m of film thickness) was used to separate different fatty acid methyl esters. During this process helium was used as a carrier gas with 1.5 mL/min flow rate. The column temperature was adjusted on 120–300°C at the rate of 10°C/min. The volume of cocklebur crop biofuel sample 0.1 × L in chloroform as a solvent with the 1:10 split ratio was introduced through split mode. The range for scanning was set as m/z 50−550 of mass spectrometer with electron impact (EI) and ionization mode (Wong et al., 2008).

#### FT-IR, ^1^H, and ^13^C NMR Spectroscopic Studies

In the proposed analytical techniques and its application in biofuel study, FT-IR spectroscopy was performed through Model FTS3000MX Bio-Rad Excalibur, in the range of 400−4000 cm^–1^ for studying the cocklebur crop biofuel structural composition. The resolution was 1 cm^–1^ and 15 scans (Knothe, 2000; Haider and Qaiser, 2018). Through spectrometer Avan CE-300 MHz equipped with probes 5mm BBO, at 7.05 T both the ^1^H and ^13^C NMR spectroscopes were conducted. As internal standard the tetramethylsilane solvent and deuterated chloroform for authentication were used. With 30° pulse duration, a 1.0 scans and 8 scans recycle delay, ^1^H NMR (300-MHz) spectrum was noted.

With 30° pulse duration, 1.89 scans and 160 scans recycle delay the ^13^C NMR (75 MHz) spectrum was recorded. Mathematically, ^1^H NMR spectroscopy was also used to calculate the conversion percentage of cocklebur plant triacylglyceride to fatty acid methyl esters using equation 3 (Knothe, 2000; Haider and Qaiser, 2018).

(3) $$PercentageofBiofuel,C=100\times 2AMe/3ACH{}_{2}$$

Where, C = Conversion percentage of oil to biofuel;

AMe = Integration value of the methoxy protons in biofuel;

*ACH*_2_ = *Integration value of* α*-methylene protons in biofuel.*

#### Elemental Analysis of Cocklebur Crop Biofuel by Using AAS Spectrophotometer

Atomic Absorption Spectrophotometer (AAS) was applied to determine the amount and distribution of various elements present in cocklebur crop biofuel. To prepare the sample for analysis perchloric acid, sulfuric acid and nitric acid were taken in ration of 0.5:1:5, correspondingly. After the proposed mixture, in the conical flask 0.25 gm of testing sample was taken. At hot plate the conical flask was placed and up to the appearance of white fumes it was heated. Then the sample was taken-off from the hot plate and added 10−15 drops of distilled water for temperature lowering of the solution. Then the sample was transferred to another 50 mL container and by the addition of distal water the volume was raised up to 50 mL, then the sample was filtered through Whatmann No. 42 filter paper for the removal of impurities. This filtered and purified sample was stored for atomic absorption spectroscopic analysis using equation 4 (Shamaima et al., 2018).

E⁢l⁢e⁢m⁢e⁢n⁢t⁢s⁢C⁢a⁢t⁢i⁢o⁢n⁢i⁢n⁢C⁢o⁢c⁢k⁢l⁢e⁢b⁢u⁢r⁢C⁢r⁢o⁢p⁢B⁢i⁢o⁢f⁢u⁢e⁢l

(4) =(p⁢p⁢m⁢i⁢n⁢f⁢i⁢l⁢t⁢r⁢a⁢t⁢e-b⁢l⁢a⁢n⁢k)/W×d⁢i⁢l⁢u⁢t⁢i⁢o⁢n⁢f⁢a⁢c⁢t⁢o⁢r×A

*Where*; A = Total volume of filtrate (mL);

W = Weight of biodiesel sample.

## Results and Discussion

### Cocklebur Crop Oil Extraction and FFAs Content Determination

The implementation of two techniques used for oil extraction was important to compare the results of applied methods with those of universal method reported in literature (Syers et al., 2007; Gui et al., 2008; Singh and Dipti, 2010). European Union has specified the soxhlet extraction method for oil extraction in seeds. While, the two technologies used in this work was very simple, easy to handle and coast effective (Linda, 2009; Ravi and Sastry, 2009). In this work, the seeds oil expression was reported 37.2%, accordingly. Before the synthesis of biofuel, FFAs content in the cocklebur crop oil sample was checked using the acid base titration method. The FFAs content was found 0.47 mg KOH/g, fall within the range of listed limit. According to the research work of Anggraini and Wiederwertung, if the FFAs content in oil is more than 3%, the conversion efficiency of oil to biodiesel will be decreased gradually (Archana and Khale, 2011).

### ZnO Nano-Particle Structural Analysis

**FIGURE 2:**
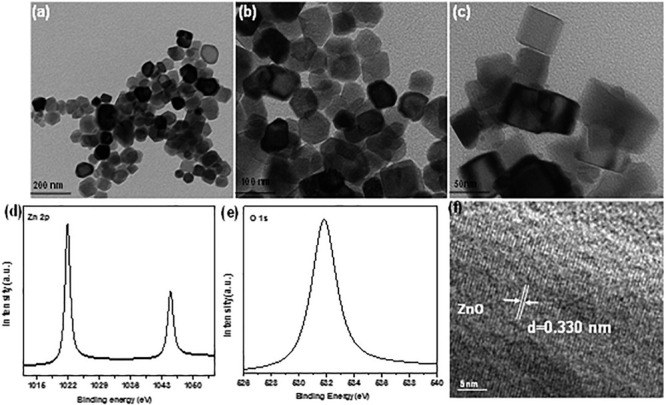
TEM micrograph images of panels **(a–c)** ZnO nano boxes **(d,e)** XPS of Zn 2p and O 1s **(f)** HRTEM of ZnO.

ZnO nano-particle was characterized by TEM and the micrograph images (Figures 2a–c), presented a rectangular boxes at various magnifications. The size of box lies under the range of 50−60 nm (del Río et al., 2016; Mesch et al., 2016; Tel-Vered et al., 2016). The surface valance states of elements present in ZnO nano boxes sample was investigated by XPS machine as shown in Figures 2d,e. The lattice spacing was observed by HRTEM image up to 0.330 nm, corresponding the plane of ZnO (002) as shown in Figure 2f. The UV-vis DRS of ZnO nano boxes displayed in Figure 2a shows the ZnO nano boxes absorption edges bandgap 3.12, as shown in Tauc plot (Figure 2b; Mitsudome et al., 2008; Silva et al., 2016).

**FIGURE 3:**
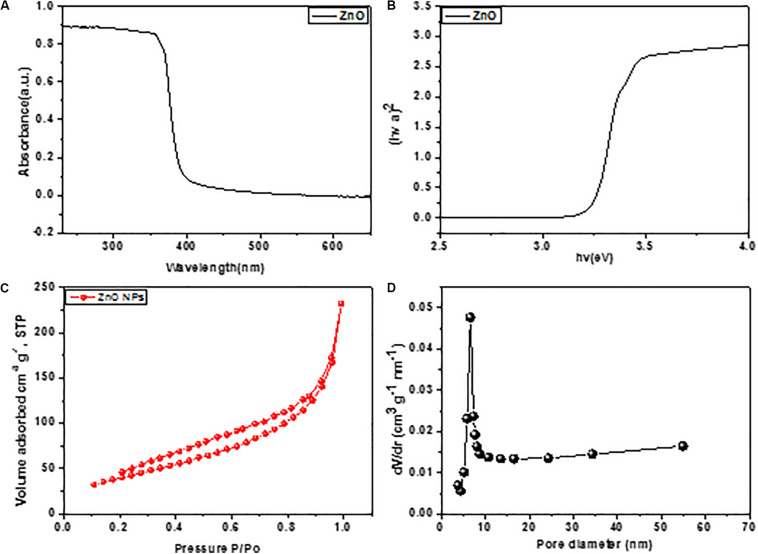
**(A)** UV-vis spectra of ZnO and **(B)** ZnO with the corresponding plots of [F(R∞)hv]^2^ vs. hv. **(C,D)** N_2_ adsorption–desorption isotherms and pore size distributions of ZnO nano boxes.

Furthermore, to know the surface status of ZnO nano boxes, N_2_ adsorption-desorption sequence was adjusted. In Figure 3C, clearly showed the N_2_ adsorption/desorption isotherms and pore size of ZnO nano boxes. The proposed finding presented in Figure 3C of isotherms recognized a particular IV hysteresis loop, under the definition of IUPAC. The locking of liquid N_2_ and delayed evaporation in desorption isotherm showed the compound behavior similar to H3 hysteresis loops, implying the hierarchical pores. It was cleared that ZnO nano boxes was having a largest surface area 65.3 m^2^ g^–1^. The position of pore distribution reconfirmed the Barrett Joyner Halenda theory about the pore characteristics of ZnO nano boxes as shown in Figure 3D. In Figure 3D, the pore size of HNTs distributed in between 12 nm, corresponding to the cylindrical hole of nano box and accumulation hole, respectively.

**FIGURE 4:**
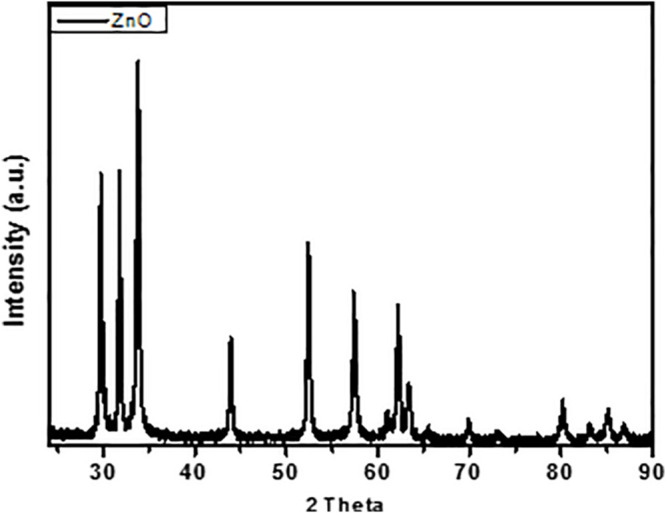
XRD patterns of as-prepared ZnO nano boxes.

The synthesized particle was characterized by XRD technique to know the structure status of ZnO nano boxes as shown in Figure 4. The absolute diffraction peaks indexed to the tetragonal phase of ZnO, PDF 65-3411, confirming the pure crystallization of ZnO nano boxes. Along with, no extra peaks of the phases were detected, rectify the particle synthesis purity (Wong et al., 2008; Begum et al., 2016; Shi et al., 2016).

### Biofuel Synthesis

The factors that greatly influenced the yield of biofuel are methanol oil molar ratio, catalyst concentration, temperature and reaction time. In the present research work, the effects of different variables were checked on the production of non-edible oil of energy crop cocklebur to know the optimum protocol for maximum conversion of triacylglyceride to fatty acid methyl esters. The following variables were checked and reported the findings below in detail.

#### Effect of Oil-Methanol Molar Ratio on Conversion Yield

**FIGURE 5:**
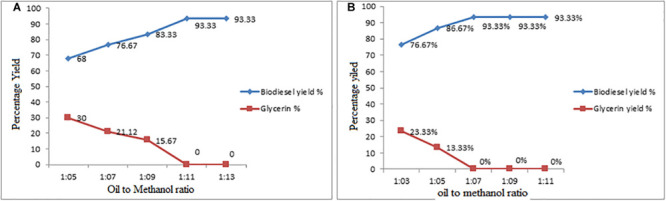
**(A,B)** Effect of Oil: Methanol Molar Ratio on Esterification and Transesterification Steps.

The main role of catalyst in reaction kinetics is to reduce the activation energy. One of the major parameters the oil-methanol molar ration is considered that controls both the esterification and transesterification reaction and its impact on conversion yield (Shamaima et al., 2018). In the present research work, the oil-methanol molar ratios were designed varied from 1:5, 1:7, 1:9, 1:11, 1:13 to 1:3, 1:5, 1:7, 1:9, 1:11 in the esterification and transesterification reaction, respectively. The reported results shown in Figures 3A,B, indicate that the maximum conversion yield were achieved at 1:11−1:7 oil-methanol molar ration in esterification and the transesterification, respectively. It was observed in the series of experiments, which with 1:11−1:7 oil-methanol molar ratio in esterification and transesterification reaction were achieved the maximum conversion of biofuel yield 93.33% Figures 5A,B. The findings reported in the present work found similar with work reported by Rahmadhas, Worapun etc. (Ronald et al., 2012; Mofijur et al., 2019).

As known to us that for the transesterification the oil to alcohol stoichiometric molar ratio is 3:1 and this is the reversible reaction. Therefore, to upturn the miscibility and to enhance the contact between the alcohol molecule and the triglyceride higher molar ratios are required. It is well known to us, the molar ratio should be higher than that of the stoichiometric ratio for shifting a reaction toward completion (Anggraini and Wiederwertung, 1999).

To break the glycerin fatty acid linkages during the transesterification presence of sufficient amount of methanol is needed. However, methanol excess should be avoided because increase in the oil/methanol molar ratio beyond 1:6 neither raises the yield of product nor the contents of ester. However, it marks the recovery process of ester difficult and increased the cost. According to the Leung and Guo (Abd Rabu et al., 2012) the methanol contains hydroxyl group which is polar and thus it may acts as an emulsifier causing emulsification. As a result the separation of water from ester becomes hard. According to the Miao and Wu (Worapun et al., 2012) during the process of biofuel synthesis the addition of methanol in large quantity (70:1 or 84:1) reduced the parting of ester and glycerol phases.

#### Effect of Catalyst Concentration on Conversion Yield

On the biodiesel yield there is a significant effect of the concentration of catalyst (Ullah et al., 2014). Using catalyst concentrations ranging from 0.10, 0.15, 0.20, 0.25, 0.30%, respectively the cocklebur crop oil esterification reaction was carried out. It was observed in the series of reactions, that at 0.3% catalyst (w/w) the maximum conversion yield was obtained. Consequently, the percentage of biofuel yield increased with increasing of H_2_SO_4_ quantity. At low temperatures, the kinetic energy of fat molecule was reduced thus needed more number of catalyst molecules to boost up the reaction (Ashok et al., 2017).

**FIGURE 6:**
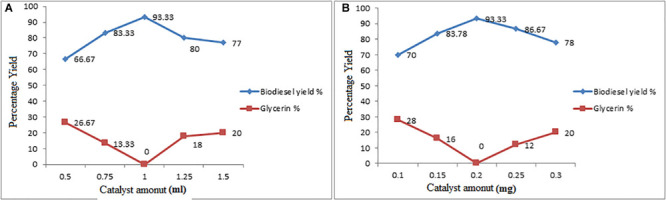
**(A,B)** Effect of ZnO Catalyst Concentration on Esterification and Transesterification Steps.

Similarly, in transesterification reaction, the maximum conversion yield was observed with optimum catalyst concentration at 0.20%, as shown in Figures 6A,B. It was observed that primarily the yield increased by increasing H_2_SO_4_ concentration up to 1 mL and then gradually decreased the yield by increasing up to 1.50 mL. Similarly, in transesterification reaction, methyl ester yield was increased with the increases in ZnO concentration up to 0.20 mg and then gradually decreased by increasing the catalyst concentration. This is because of the higher catalyst concentration cause soap formation. The formation of soap was due to the ester dissolution into free glycerol. The formation of soap due to emulsification was also reported by Bojan and Leung (Miao and Wu, 2006). Somewhere else it was reported that with the catalyst concentration increment decrease in the methyl ester yield occur because of soap production because of high catalyst amount, this is because of the increased viscosity of reactants thus it dropped the yield (Knothe et al., 2003; Bojan and Durairaj, 2012).

To obtained maximum and quality biodiesel during ester exchange reaction the catalyst stability was also checked. But, it has been observed it was noted during the first three stages of reaction series that the performance remains consistent however in the fourth stage of reaction decrease in the yield of biofuel to some extent was observed. Molina (Ullah et al., 2015a) observed in the initial two stages 95% yield, but in the third stage of reactions the yield was decreased.

#### Effect of Temperature on Conversion Yield

The temperature effect on reaction at various levels starting from 50, 55, 60, 65, to 70°C on esterification and transesterification reaction was applied to know the optimum conversion yield. The maximum level of temperature during reaction enhance the reaction kinetics, reduces the duration of reaction and proportionally alter the change in yield (Ullah et al., 2015b; Teshome and Karthikeyan, 2019).

**FIGURE 7:**
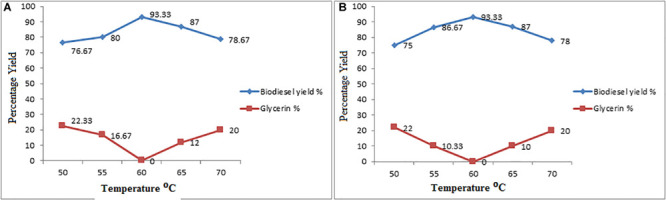
**(A,B)** Effect of Reaction Temperature on Esterification and Transesterification Steps.

The temperature effect on reaction during conversion on cocklebur crop biofuel yield was checked with various temperature level during reaction, using the esterification followed by transesterification process. In the reported work data, findings shows in Figures 7A,B the effect of temperature on biofuel yield at various temperature levels, ranging from 50, 55, 60, 65, 70°C, respectively. With increasing temperatures from 50 to 60°C, increased the conversion yield up to 93.33%, and decreases the yield by increase of temperature up to 70°C. The reported data found similar to the research work reported by Uzan, Phan, Zhang etc. (Agus et al., 2014; Baskar et al., 2019; Mariem et al., 2019). The reduction flow in conversion yield with elevated temperature is possible due to high miscibility, which reduces the phase separation and yield. The work reported by Leung and Guo shows that higher the temperature proceeds negative impact on yield, whereas, shows a positive effect for those oils who has in viscous state.

#### Effect of Reaction Time on Biofuel Conversion Yield

The conversion rate of vegetable oils into biofuel increases by increasing of reaction time. The process of reaction initially slows due to the mixing and dispersion of alcohol with oil, and gradually increases with the increasing of temperature.

**FIGURE 8:**
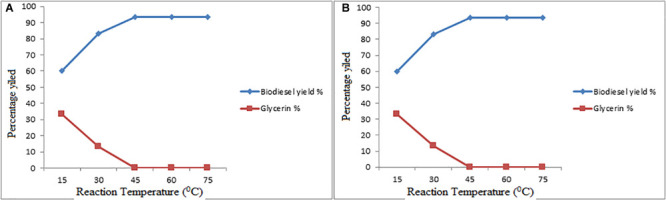
**(A,B)** Effect of Reaction Time on Esterification and Transesterification Steps.

In the present research work, the effect of reaction time on biofuel yield was checked as shown in Figures 8A,B. The reaction time with fixed interval duration was adjusted, started from 45, 90, 135, 180, 225 min, respectively. Over all, in the series of experimental data, the maximum conversion yield was achieved at 60 min (93.33%). Similarly, in the transesterification reaction, the time was adjusted from 15, 30, 45, 60, 75 min, respectively. In this reaction, the maximum conversion yield of biodiesel was achieved at 45 min. Considering the above findings, the same results of biodiesel yield under the same condition were reported by Cyali and Supardan (Uzun et al., 2012).

### Fuel Properties Analysis of Cocklebur Crop Biofuel

The Physico-chemical characteristics of biofuel is evaluated under the specified international ASTM and EN standards (Phan and Phan, 2008; Moazeni et al., 2019). For the engine parts the biodiesel quality is most important. To check out the fuel quality, various international standards are specified for the fuel quality control. The following fuel properties of cocklebur crop biofuel were investigated and compared to standards of ASTM D 6751- 06 & EN 14214 as shown in Table 1. In the diesel engine the key issue related to the pure vegetable oils use in diesel engines as fuel is due to its high viscosity particularly during its ignition process. The high viscosity results in fuel poor atomization, incomplete combustion, fuel injectors, ring carbonization and accumulation of fuel in the lubricating parts etc. (El Diwani et al., 2009).

**TABLE 1:** Comparative Fuel Properties Analysis of Energy Crop Cocklebur Methyl Esters “B100”.

**Work parameters**	**Density (15°C, g cm^–3^)**	**Kinematic viscosity at 40°C, mm^2^/s**	**PP (°C)**	**CP (°C)**	**Phosphorus% (wt)**	**Acid number mg KOH/g**	**Flash point (°C)**	**Calorific value (kj/kg)**	**Sulfur (%mass)**
ASTM D6751	−	1.9−6.0	−15−16	−3−12	0.001	0.50 max.	130 min	−	0.05
EN 14214	860−900	3.5−5.0	−	−	−	0.50 max.	120 min	−	0.01
Petro−diesel	859.0	3.14	−35, −15	−15−5	−	−	60−80	47216	0.034
Biodiesel (B100)	877.00	3.76	−9.00	+2.00	0.00	0.33	83.00	1634	0.05

Quantitatively, the fuel properties of cocklebur crop biofuel were determined and compared to ASTM D 6751 and EN 14214 standards in contrast to petro-diesel. The following fuel tests of cocklebur crop biofuel: Kinematic viscosity at 40°C (St), Density at 40°C (Kg/L), Flash Point (°C), Cloud Point (°C), Pour Point (°C), Calorific Value Kj/Kg, Total acid no. mg KOH/g, Distillation @ 90% recovery (°C), Sulfur contents (%) and Cetane index were determined and compared with the ASTM D 6752 & EN 14214 standards, respectively.

#### Acid Value

It is the measure of the free acids contents in the sample of proposed synthesized biofuel. The Acid value in the synthesized cocklebur crop biofuel was found 0.33 mg KOH/g. The Acid value investigated was found less than the standard range (0.5 mg KOH/g) by EN 14214. The Acid value reported in this work match with those reported by Felizardo, Shalaby, Predojevic, Skrbic etc. in the Sunflower, Maize and Canola (Abd Rabu et al., 2012; Worapun et al., 2012; Ullah et al., 2014; Ashok et al., 2017). The purification of biofuel also affects the acid values. The Acid value of biofuel was determined by means of silica gel ranging in 0.1 mg KOH/g to 0.39 mg KOH/g, while biofuel treated with phosphoric acid ranging between 0.2 mg KOH/g to 0.34 mg KOH/g, respectively. The purification of biofuel with hot distilled water increased the acid values probably from 0.23 mg KOH/g to 0.8 mg KOH/g, accordingly (Holser and O’Kuru, 2006; Henok et al., 2019).

#### Kinematic Viscosity

The degree of fuel internal fluid friction to its pour is called viscosity. About fuel atomization of fuel, and fuel distribution kinematic Viscosity is the important quality control parameter. Injector atomization and lubrication is affected by fuel viscosity. When the fuel has low viscosity, it results in less lubrication for fuel injection pumps precision fit, which leads to increased wear or leakage of the injection pump. This leakage may increase the power loss for the engine; while, with high viscosity the injection pump cannot supply enough fuel to the pumping chamber, ultimately it results in the engine power loss. Furthermore if the viscosity of fuel is high on injection it forms droplets of larger size, this may leads to poor fuel combustion, and high smoke exhaustion. For the direct use of vegetable oil as fuel in engine high viscosity and low volatility are the two main hurdles (Felizardo et al., 2006; Predojevic, 2008; Rao, 2011; Shalaby and El Gendy, 2012; Sheikh et al., 2013). In the biofuel preparation, high viscosity of vegetables oil negatively affects the conversion efficiency and restricts the mixing of substrates.

The measured Kinematic viscosity range in the cocklebur crop biofuel was found 3.76 mm^2^/s at 40°C. This investigated value was found similar with the limit of ASTM D 6752 stander as shown in Table 1, while, slightly found higher than the EN 14214 range. Comparatively, the cocklebur crop biofuel Kinematic viscosity seems closer to Petro-diesel as shown in Table 1.

#### Density

In airless combustion systems this property is of great significance as it affects the fuel atomization efficiency. It also affects the fuel spray breaking-up from the injector. Therefore, with the increase of density of fuel by mass more fuel is injected. As we know that the injection system of fuel works on a volume metering system, thus the fuel having less content of energy per liter may results in the generation of less peak power; but the high density of biofuel compensates the lower energy content (No, 2011). In this work, the density of cocklebur crop biofuel was found 0.8770 g/cm^3^, which found under the range of EN 14214 standards (Table 1).

#### Calorific Value

In the selection of a quality fuel it is an important parameter. Due to high oxygen contents the biofuel caloric value is usually lesser than the diesel fuel (Anguebes et al., 2019). The finding data presented in Table 1 showed that the calorific value of cocklebur crop biofuel is comparatively lower than the petro-diesel fuel.

#### Flash Point

The temperature at which fuel ignites when it is exposed to flame is called flash point. It is the result of the formation of homogenous mixture of fuel vapor and air above the fuel surface. In fuel storage and handling it is an important parameter (Perkins and Peterson, 1991; Sara et al., 2020). According to the standard of the EN 14214 the flash point for biofuel is higher than 120°C; on the other hand the ASTM D 6751-02 specifies its range to be under 130°C. The methanol content of biofuel affects the flash point. With the increase of 0.5% methanol content in biofuel leads up to 50°C decrease of the biofuel flash point. In the cocklebur crop biofuel sample, the flash point was reported 83°C which fall in the range of EN 14214 & ASTM D 6751-02 standards. The flash point reported in the available literature showed that the flash point should be within the range of 160−202°C, respectively (Srivastava and Prasad, 2000; Encinar et al., 2005; Ullah et al., 2015d).

#### Cetane Number

The biofuel ignition performance can be the calculated from its cetane number. If the cetane number of a fuel is high then the ignition delay will be shorter. In present work the biodiesel cetane number was 50, which is higher than the range of the standard of ASTM D 6751-10. But was lower than the standard fixed by EN 14214 (Table 1). Over all, for its quality conformation, a small amount of nitric acid Iso-octyl rise up to the exact range (Dias et al., 2008; Refaat et al., 2008). It is clear from the above narration that the unsaturated degree of fatty acid is inversely proportional to the cetane number.

#### Cloud Point (CP), Pour Point (PP), and Cold Filter Plugging Point (CFPP)

For low temperature applications of fuel cloud point and pour point are the two important parameters. When the fuel is cooled and wax first becomes visible that point of temperature is cloud point. That minimum temperature at which biofuel can still flow is called as pour point. The maximum values for pour point and cloud point in the synthesized biofuel sample were measured 2°C and −9°C, as shown in Table 1. There is no limit is given by the ASTM D 6751 standard; but for both cloud and pour point rather a “report” is specified. According to the ASTM D 6371 standard Cold Filter Plugging Point test was conducted. The value for CFPP was reported in the range of −4°C and −9°C, respectively as shown in Table 1 (Theocharis et al., 2019).

#### Sulfur and Phosphorus Contents

Biofuel has less amount of sulfur as compare to fossil fuel therefore emits less sulfur dioxide as compare to fossil fuels. In the present study, the sulfur content in cocklebur crop biofuel was found 0.0047 ppm, whereas, the amount specified in ASTM D 4294 is 0.05 ppm, respectively. According to Kumar et al., sulfur content is an important feature in terms of reduction of sulfur dioxide that from the exhaust emissions. The sulfur was not detected in elemental analysis studied by Wang, whereas, Kumar found the Sulfur content in the range of 11 ppm, which is higher than the level found in Petro-diesel (Knothe, 2009; Sultana et al., 2016). According to the ASTM D 6751 and EN 14214 standards, a specific range are given for the concentration of elemental contents. Similarly, the phosphorous content in cocklebur crop biofuel is negligible. Thus, the cocklebur crop biofuel found free of sulfur and phosphorus contents and to be considered an environment friendly fuel (Zhang et al., 1988).

### Characterization and Quantification of Cocklebur Crop Biofuel via FT-IR, NMR, and GC-MS Analysis

To define the fatty acid methyl esters profile and chemical structure of cocklebur crop biofuel, various analytical techniques i-e FT-IR, NMR and GC-MS spectroscopes were performed. The applications of various analytical techniques were applied do determine and confirm in general the quantification and characterization of oil conversion to biofuel (Wang et al., 2011).

#### FT-IR Spectroscopic Analysis

**FIGURE 9:**
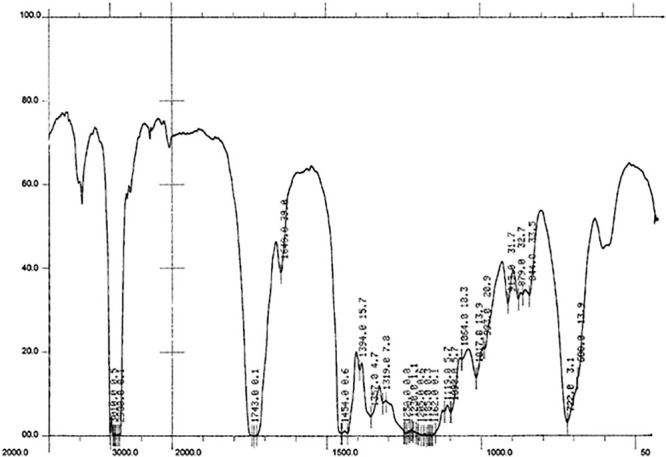
FT-IR Spectrum Representing 12 Major Absorption Peaks in Energy Crops Cocklebur Methyl Esters.

The application of FT-IR spectroscopy was designed to monitor the undergoing transesterification reaction. During the analysis, different bonds vibration were determined in the cocklebur crop biofuel as shown in Figure 9.

In FT-IR the carbonyl group locus is sensitive to the molecular structure and to substituent effects (Dias et al., 2008). At 1737 cm^–1^ the methoxy carbonyl group was obtained while on 1015 cm^–1^ the ether (C-O) linkage was achieved. For the confirmation of biofuel production the presence of these bonds is the mark. For methylene 2855 cm^–1^ and 1167 wavenumber bands (cm^–1^) for the stretching and banding were obtained, and for C-H aromatic the wavenumber bands (cm^–1^) 3005 was obtained. Likewise, at 1061 cm^–1^ the alcohol stretching wavenumber bands (cm^–1^) was obtained and at 1433cm^–1^ the C = C aromatic wavenumber bands (cm^–1^) was found (Table 2).

**TABLE 2:** List of Functional Groups Observed in Energy Crop Cocklebur Methyl Esters using FT-IR spectroscopy.

**Sr./No.**	**Functional Groups**	**Observed Peaks**
01	Methoxy carbonyl	1737
02	Ether	1015
03	Methylene (Stretching)	2855
04	Methylene (Banding)	1167
05	C-H (Aromatic)	3005
06	Alcohol (Stretching)	1016
07	Aromatic (C = C)	1433

The FT-IR spectroscopic study has been done to approve the production of biofuel and its bonds vibration. At 1730−1750, carbonyl wavenumber bands (cm^–1^) was found and at 1000−1300 cm^–1^ C-O, wavenumber bands was obtained, these are the two main characteristic wavenumber bands (cm^–1^) for the recognition of ester. In present study for cocklebur crop biofuel at 1737 cm^–1^ and 1015 cm^–1^ the carbonyl (νC (=O) and ester (C-O) wavenumber bands (cm^–1^) were found correspondingly. While at 1167 cm^–1^ and 2855 cm^–1^ the wavenumber bands for methylene banding and stretching were observed, respectively (Figure 9). Likewise, at 1433 cm^–1^, 1061 cm^–1^, and 3005 cm^–1^ the wavenumber bands of C = C aromatic, alcohol stretching and C-H aromatic were obtained, respectively (Kumar, 2013; Ullah et al., 2014).

#### ^1^H and ^13^C NMR Spectroscopy

**FIGURE 10:**
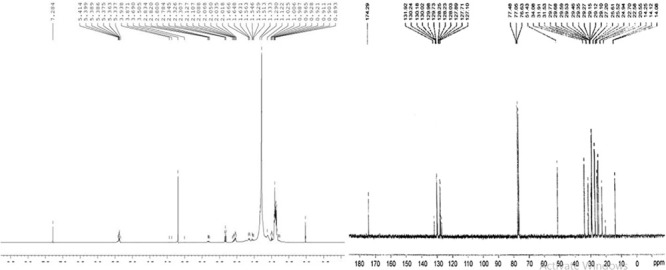
**(A,B)** ^1^H NMR and ^13^C NMR Spectrum Showing Various Absorption Peaks in Energy Crop Cocklebur Methyl Esters.

^1^H NMR spectroscopy was conducted for the characterization of cocklebur crop biofuel (Figure 10A). The transformation of triglyceride to fatty acid methyl esters was confirmed from the characteristic single of methoxy protons (–OCH_3_) at 3.50 ppm for. Similarly other important signals such as at 2.01−2.83 ppm for α-methylene protons (α–CH_2_), at 0.89−0.98 for terminal methyl protons (–CH_3_), at 1.00−1.66 for β-methylene protons (β-CH_2_) and at 5.33−5.41 ppm for olefinic hydrogen (–CH_2_) were observed in a triplet form. In the biofuel of the cocklebur crop different fatty acid methyl esters signals confirm the formation of biofuel (Table 3).

**TABLE 3:** Reported Fatty Acid Methyl Esters in Energy Crop Cocklebur Biofuel, With Retention Time and %tage using GC/MS.

**Sr. No.**	**Identified fatty acid methyl esters**	**Formula of FAME**	**Retention time**	**%Age of compounds**
01	Hexanoic acid methyl ester	C_6_:0	2.934	0.001
02	Caprylic acid methyl ester	C_8_:0	4.739	0.001
03	Capric acid methyl ester	C_10_:0	6.476	0.002
04	Lauric acid methyl ester	C_12_:0	8.077	0.008
05	Tridecanoic acid methyl ester	C_13_:0	8.673	0.001
06	Myristic acid methyl ester	C_14_:0	10.168	0.034
07	Pentadecanoic acid methyl ester	C_15_:0	11.627	0.012
08	Palmitic acid methyl ester	C_16_:0	13.424	2.251
09	Palmitoleic acid methyl ester	C_16_:1	13.878	0.155
10	Margaric acid methyl ester	C_17_:0	15.5	0.021
11	Heptadecenoic acid methyl ester	C_17_:1	15.943	0.016
12	Stearic Acid methyl ester	C_18_:0	17.897	1.355
13	Oleic Acid methyl ester	C_18_:1c	18.375	11.872
14	Elaidic acid methyl ester	C_18_:1n9t	18.506	1.133
15	Linolenic acid methyl ester	C_18_:2c	19.659	16.419
16	Octadecadienoic acid methyl ester	C_18_:2t	19.817	0.049
17	Linolenic acid methyl ester	C_18_:3n3	21.743	9.453
18	Arachidic acid methyl ester	C_20_:0	24.661	0.96
19	11-Eicosadienoic acid methyl ester	C_20_:1c	25.315	8.011
20	11, 14-Eicosadienoic acid methyl ester	C_20_:2c	26.948	0.568
21	Heneicosanoic acid methyl ester	C_21_:0	28.368	0.007
22	11, 14, 17-Eicosanoic acid methyl ester	C_20_:1n9	29.259	0.091
23	Behenic acid methyl ester	C_22_:0	31.994	0.834
24	Erucic acid methyl ester	C_22_:1n9	33.91	42.034
25	13, 16-Eicosadienoic acid methyl ester	C_22_:2c	35.03	0.751
26	Tricosanoic acid methyl ester	C_23_:0	35.03	0.02
27	Lignoceric acid methyl ester	C_24_:0	38.08	0.246
28	Nervonic acid methyl ester	C_24_:1	38.611	1.166

Through the transesterification process the percentage conversion of cocklebur crop oil to biofuel was determined by ^1^H NMR (Christie, 2003) α-carbonyl methylene protons (2.00−2.80 ppm) and methyl group in biofuel (3.49–3.86 ppm) are the two concerned signals. In this research work, the maximum transformation proportion of cocklebur plant oil into analogous methyl esters through equation 3 was found 93.33%.

The cocklebur crop biofuel chemical structure and its spectrum were studied through ^13^C NMR spectroscopy (Figure 10B). The characteristic signals of ester carbonyl group (–COOR) was appeared at 174.26 and for methoxy carbon (–OMe) appeared at 51.35 ppm in the ^13^C NMR. The position of un-saturation (C = C) was confirmed from the signals obtained at 127.23 and 131.94 ppm in the cocklebur crop biofuel. Similarly the signals 14.03−14.23 ppm was obtained for methyl groups (–CH_3_) terminal carbon and the 25.34−34.11 ppm was obtained for long carbon chain ethylene carbons (–CH_2_-).

#### Determination of Metals Concentration in Cocklebur Crop Biofuel

**FIGURE 11:**
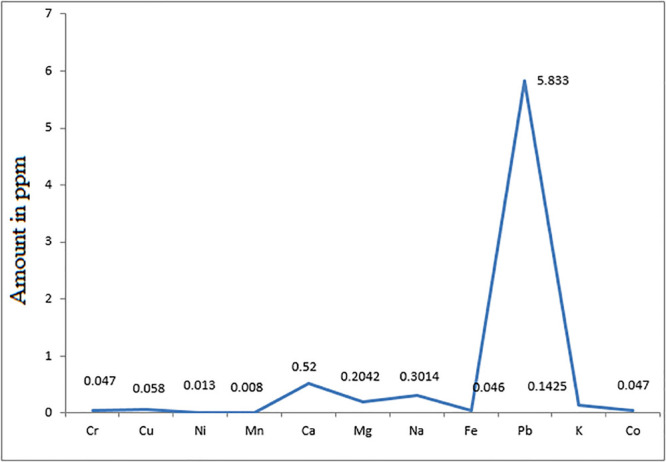
Concentration and Distribution of Various Elements in Energy Crop Cocklebur Methyl Esters.

In this study, the concentration of various metals in cocklebur crop synthesized biofuel (Figure 11) were investigated and compared with ASTM standard. The purpose of metal detection is to avoid the engine deposition, environmental pollution and better combustion. The concentrations of various metals were determined by using the Atomic Absorption Spectrophotometer (Varion GTA 120, United States) (Christie, 2003). The concentration of each element was determined and calculated in ppm unite.

In biofuel the presence of metals is detrimental, because it can leads to several problems such as degradation of biofuel, engine corrosion and pollution (Ramadhas et al., 2005). The quantities of the following elements need to be controlled in biofuel sodium (Na), potassium (P) and phosphorus (P) that mainly come from raw materials. The metallic concentration in cocklebur crop biofuel was compared with petro-diesel. The results clarify that the concentrations of metals present in cocklebur crop synthesized biofuel were comparatively lower than the limit of petro-diesel.

The presence of metals such as calcium, magnesium, potassium, and sodium in biofuel result in engine deposition, deposition in fuel pump, deposition in injector, piston, ring wear, and plugging of filter (Schober and Mittelbach, 2005). The finding data of calcium, magnesium, potassium, and sodium in concentration were found 0.52 ppm, 0.2042 ppm, 0.3014 ppm, and 0.1425 ppm, respectively in the cocklebur crop biofuel sample. The concentration range of these metals present in petro-diesel is 213.3, 868.3, 35.6, 21.4 ppm, which are comparatively higher than the proposed synthesized biofuel. The maximum possibilities of sodium and potassium in biofuel are 0.5 ppm, while, the range of potassium may exceeded up to 10 ppm.

Similarly, the rate of gum formation is more when there is more concentration of copper and iron as compared to nickel and zinc. The concentration of Fe and Ni in cocklebur crop biofuel was reported 0.046, 0.013 ppm, which fall down than the limit fixed for petro-diesel (Figure 11). The burning of lead causes deposition in engine that causes corrosion of engine. The concentration of lead in cocklebur crop biofuel was found 5.83 ppm that remained lesser from fossil-diesel. The heavy metals emanation like lead from automobiles is the key disease source in men (Korn et al., 2007; McCormick et al., 2007; Teixeira et al., 2007). The metal analysis described in cocklebur crop biofuel showed that it is safe as alternative to mineral diesel for environment and have a better compatibility with the diesel engine as well.

#### GC-MS Analysis

The chemical composition and quantification of cocklebur crop biofuel was analyzed through GC-MS study. In the investigated data, the GC chromatogram showed 28 peaks. From the library match software (NO. NIST 02) the peaks obtained for various fatty acid methyl esters were confirmed. With the help of mass spectrometric analysis and retention time data the fatty acid methyl esters identity was primed (Table 3). Through EI ion source the mass spectrum was obtained. The chromatogram analysis showed that Oleic Acid (C18:1c), Linolenic acid (C18:2c), Linolenic acid (C18:3n3), 11-Eicosadienoic acid (C_20_:1c) and Erucic acid methyl esters (C_22_:1n9) were the major fatty acid methyl esters found in cocklebur crop biofuel. During the analysis, 15 saturated and 13 unsaturated fatty acid methyl esters were identified in the proposed synthesized biofuel taster, respectively.

**FIGURE 12:**
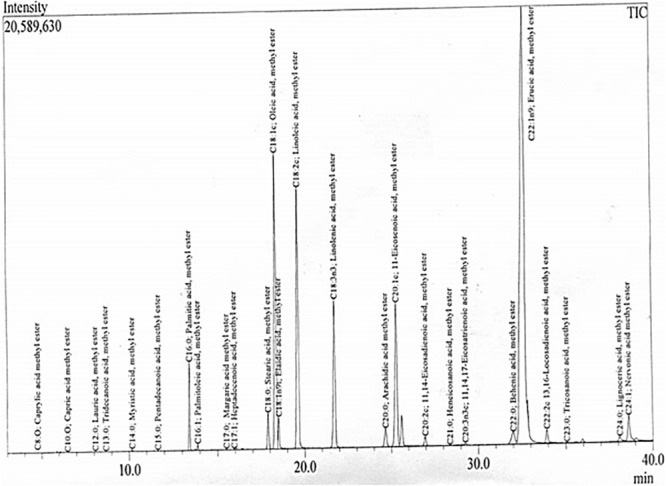
GC/MS Chromatogram Showing 28 Major Methyl Esters in Energy Crop Cocklebur Biofuel Sample.

The fatty acid methyl esters identification was done through GC-MS spectroscopy with the electron ion mode. Total twenty eight major peaks have been obtained in the cocklebur crop biofuel as shown in Figure 12. Through library match software (NO. NIST 02) the peaks for the various fatty acid methyl esters were recognized. The GC-MS examination of the biofuel produced from cocklebur crop was summarized in Table 3. The GC-chromatogram experimental run data was done at 300°C for 40 min with 1:11 oil-methanol tells the existence of saturated and mono-unsaturated fatty acid methyl esters major proportion. The eluted components retention time helps in identification, while the GC-fragmentation patterns were used for the confirmation of various fatty acid methyl esters. The GC-MS data demonstrated that the cocklebur crop biofuel was mainly composed of different FAMEs as shown in Figure 12. In the study showed that Oleic Acid (C18:1c), Linolenic acid (C18:2c), Linolenic acid (C18:3n3), 11-Eicosadienoic acid (C20:1c) and Erucic acid methyl esters (C22:1n9) are the major esters and composed of 89% of the total fatty acid methyl esters occur in cocklebur crop biofuel tester.

The study reported by Kouame shows that various methyl esters; Caprylic acid, Eicosanoic acid and Behenic acid found in Jatropha and Soybean biofuel. Similarly, the Palmitic acid, Palmitoleic acid, Stearic Acid, Oleic Acid, Linolenic acid, and Arachidic acid methyl esters were consequently reported by Predojevic in Jatropha biofuel. The literature study regarding the fatty acid methyl esters composition of different plant species biofuel showed that different researchers reported 8−16 types of fatty acid methyl esters (Kouame, 2011; Nzair et al., 2013). This study first time, reporting total 28 numbers of different fatty acid methyl esters in the synthesized sample of cocklebur crop as shown in Table 3.

## Cocklebur; Novel Non-Edible Energy Crop, A Feasible Source of Biofuel

Non-edible energy crops oil are not used in for human as a food because of the some toxic compound presence. For example, linseed oil has not been used for cooking purpose because at high temperature, omega fatty acids have broken into toxic compound. Therefore the selection of non-edible oil yielding plant species for biofuel production provide a smooth background over those resources which are being used as a feedstock for biofuel production. In the present research work, a comprehensive research study on biofuel production show the importance of non-edible energy crops over other feedstock. These non-edible energy crops oil reduce the food related problems and various economic issues. Naturally, non-edible energy crops are resistible and can grow well in arid, semi-arid, non-fertile and adapted to adverse environmental conditions.

Moreover, non-edible energy crops are expected to use the in degraded forests, rail road’s, and irrigation canals. In this regard, the non-edible energy crops cultivation play an important role in poverty elevation of the rural areas, particularly in the energy sector. Apart from each, many researchers have concluded, that non-edible energy crops resources for biofuel production considered as a renewable and sustainable fuel. The proposed investigated energy crop/feedstock is non-edible, whereas they do not compete for food crises. The comparative studies include the selection and recommendation of most suitable plant in respect of their easily availability, cheap production, economic and commercial abundance.

## Strengths and Limitations of the Present Research Work

The feedstock used in this work is non-edible and novel. In the present research work, we were not restricted to the synthesis of biodiesel but also perform the experiments to determine the optimum conditions for maximum yield of biodiesel. Furthermore, all the physio-chemical properties of synthesized biodiesel in this work was examined in detail and found similar, according to the standards fixed internationally for qualitative biodiesel. One of the most important strength of this work is the conditions of various variables for maximum yield are very impressive. However, there was one limitation of the study. Which was we did not find any conclusion that the FFA of oil was 0.47 mg KOH/g but still we did not succeed in the synthesis of biodiesel through one step process (directly *trans* esterification) instead we followed up two step process, the esterification followed by transesterification).

## Conclusion

The finding observations indicated that cocklebur crop biofuel is a non-edible biomass and a promising source for quantitative and qualitative production of biofuel. Considering the above explanation, the biofuel produced with the optimum protocol overall met the ultimate requirements of petro-diesel. Consequently, it may be a possible alternative to petro-diesel. Keeping in view, the physio-chemical characteristics and fuel lands dissection, the cocklebur crop oil is considered as a potential non-edible oil source for bioenergy industry in general. Moreover, the non-edible novel crop economically is very cheap, available indigenously and grows easily in a variety of environment.

The experimental setup optimized for methanolysis of cocklebur crop biofuel was adjusted to 1:7 molar ratio of oil to methanol, temperature 60°C, reaction time 45 min and 0.2 gm ZnO (w/w). The proposed defined experimental set provided 93.33% yield of cocklebur crop biofuel. The synthesized biofuel tests were quit similar to the ASTM & EN standards. It is summarized that cocklebur crop biofuel is a reliable and alternative source to petroleum industry. Geographically, the environmental and land status of Pakistan is lies under the feasible cultivation of a particular crop for optimum production, that may contributed well to bioenergy industry.

## Data Availability Statement

The datasets generated for this study can be found in the National Herbarium of Pakistan, QAU, Islamabad, Pakistan.

## Author Contributions

All the authors listed above in the article authorship line have contributed equally to the proposed research project, and all those authors who are qualified accordingly the proposal preparation, laboratory work, and report compilation listed in sequence wise.

## Conflict of Interest

The authors declare that the research was conducted in the absence of any commercial or financial relationships that could be construed as a potential conflict of interest.
