# Supplementary material for: Synthesis and Structural Characterization of Biofuel From Cocklebur sp., Using Zinc Oxide Nano-Particle: A Novel Energy Crop for Bioenergy Industry
Source: Front Bioeng Biotechnol. 2020 Sep 4;8:756. doi: 10.3389/fbioe.2020.00756 (PMC7498747; doi:10.3389/fbioe.2020.00756)
Supplement: Supplementary file 1 [file Table_1.DOCX]

**Table 1: Comparative Fuel Properties Analysis of Energy Crop Cocklebur Methyl Esters “B100”**

|  | **Work**  **parameters** |  | **Density (15⁰C,**  **g cm^-3^)** | | **Kinematic viscosity at 40 ⁰C, mm^2^/s** | | | **PP (⁰C)** | | **CP**  **(⁰C)** | | **Phosphorus %**  **(wt)** | | **Acid number**  **mg KOH/g** | | **Flash point**  **(⁰C)** | | **Calorific value (kj/kg)** | | **Sulfur (%mass)** | |
| --- | --- | --- | --- | --- | --- | --- | --- | --- | --- | --- | --- | --- | --- | --- | --- | --- | --- | --- | --- | --- | --- |
|  | ASTM D6751 | ̶ ̶ ̶ | | 1.9-6.0 | | -15-16 | | | -3-12 | | 0.001 | | 0.50 max. | | 130 min. | | ----- | | 0.05 | |  |
|  | EN 14214 |  | 860-900 | | 3.5-5.0 | | ̶ ̶ ̶ | | | ̶ ̶ ̶ | | ̶ ̶ ̶ | | 0.50 max. | | 120 min. | | ----- | | 0.01 | |
|  | Petro-diesel |  | 859.0 | | 3.14 | | -35,-15 | | | -15-5 | | ̶ ̶ ̶ | | ̶ ̶ ̶ | | 60-80 | | 47216 | | 0.034 | |
|  | Biodiesel  (B100) |  | 877.00 | | 3.76 | | -9.00 | | | +2.00 | | 0.00 | | 0.33 | | 83.00 | | 1634 | | 0.05 | |

**Table 2: List of Functional Groups Observed in Energy Crop Cocklebur Methyl Esters using FT-IR spectroscopy**

| **Sr./No.** | **Functional Groups** | **Observed Peaks** |  |  |
| --- | --- | --- | --- | --- |
| 01 | Methoxy carbonyl | 1737 |  |  |
|  | 02 | Ether | 1015 |  |
|  | 03 | Methylene (Stretching) | 2855 |  |
|  | 04 | Methylene (Banding) | 1167 |  |
|  | 05 | C-H (Aromatic) | 3005 |  |
|  | 06 | Alcohol (Stretching) | 1016 |  |
| 07 | Aromatic (C=C) | 1433 |  |  |

**Table 3: Reported Fatty Acid Methyl Esters in Energy Crop Cocklebur Biofuel, With Retention Time and %tage using GC/MS**

| **Sr.**  **No.** |  | | | **Identified Fatty Acid Methyl Esters** |  | **Formula of FAME** | | | **Retention**  **Time** |  | **%age of Compounds** |
| --- | --- | --- | --- | --- | --- | --- | --- | --- | --- | --- | --- |
| 01 |  | Hexanoic acid methyl ester | | | |  | C_6_:0 | | 2.934 |  | 0.001 |
| 02 |  | Caprylic acid methyl ester | | | |  | C_8_:0 | | 4.739 |  | 0.001 |
| 03 |  | Capric acid methyl ester | | | |  | C_10_:0 | | 6.476 |  | 0.002 |
| 04 |  | Lauric acid methyl ester | | | |  | C_12_:0 | | 8.077 |  | 0.008 |
| 05 |  | Tridecanoic acid methyl ester | | | |  | C_13­_:0 | | 8.673 |  | 0.001 |
| 06 |  | Myristic acid methyl ester | | | |  | C_14_:0 | | 10.168 |  | 0.034 |
| 07 |  | Pentadecanoic acid methyl ester | | | |  | C_15_:0 | | 11.627 |  | 0.012 |
| 08 |  | Palmitic acid methyl ester | | | |  | C_16_:0 | | 13.424 |  | 2.251 |
| 09 |  | Palmitoleic acid methyl ester | | | |  | C_16_:1 | | 13.878 |  | 0.155 |
| 10 |  | Margaric acid methyl ester | | | |  | C_17_:0 | | 15.5 |  | 0.021 |
| 11 |  | Heptadecenoic acid methyl ester | | | |  | C_17_:1 | | 15.943 |  | 0.016 |
| 12 |  | Stearic Acid methyl ester | | | |  | C_18_:0 | | 17.897 |  | 1.355 |
| 13 |  | Oleic Acid methyl ester | | | |  | C_18_:1c | | 18.375 |  | 11.872 |
| 14 |  | Elaidic acid methyl ester | | | |  | C_18_:1n9t | | 18.506 |  | 1.133 |
| 15 |  | Linolenic acid methyl ester | | | |  | C_18_:2c | | 19.659 |  | 16.419 |
| 16 |  | Octadecadienoic acid methyl ester | | | |  | C_18_:2t | | 19.817 |  | 0.049 |
| 17 |  | | Linolenic acid methyl ester | | |  | | C_18_:3n3 | 21.743 |  | 9.453 |
| 18 |  | | Arachidic acid methyl ester | | |  | | C_20_:0 | 24.661 |  | 0.96 |
| 19 |  | | 11-Eicosadienoic acid methyl ester | | |  | | C_20_:1c | 25.315 |  | 8.011 |
| 20 |  | | 11, 14-Eicosadienoic acid methyl ester | | |  | | C_20_:2c | 26.948 |  | 0.568 |
| 21 |  | | Heneicosanoic acid methyl ester | | |  | | C_21_:0 | 28.368 |  | 0.007 |
| 22 |  | | 11, 14, 17-Eicosanoic acid methyl ester | | |  | | C_20_:1n9 | 29.259 |  | 0.091 |
| 23 |  | | Behenic acid methyl ester | | |  | | C_22_:0 | 31.994 |  | 0.834 |
| 24 |  | | Erucic acid methyl ester | | |  | | C_22_:1n9 | 33.91 |  | 42.034 |
| 25 |  | | 13, 16-Eicosadienoic acid methyl ester | | |  | | C_22_:2c | 35.03 |  | 0.751  0.02 |
| 26 |  | | Tricosanoic acid methyl ester | | |  | | C_23_:0 | 35.03 |  |  |
| 27 |  | | Lignoceric acid methyl ester | | |  | | C_24_:0 | 38.08 |  | 0.246 |
| 28 |  | | Nervonic acid methyl ester | | |  | | C_24_:1 | 38.611 |  | 1.166 |
